# Accidental Interruption of the Cold Chain for the Preservation of the Moderna COVID-19 Vaccine

**DOI:** 10.3390/vaccines9050512

**Published:** 2021-05-17

**Authors:** Santiago Grau, Olivia Ferrández, Elena Martín-García, Rafael Maldonado

**Affiliations:** 1 Pharmacy Department, Hospital del Mar, 08003 Barcelona, Spain; oferrandez@psmar.cat; 2 Hospital del Mar Medical Research Institute (IMIM), 08003 Barcelona, Spain; elena.martin@upf.edu; 3 Laboratory of Neuropharmacology-Neurophar, Department of Experimental and Health Sciences, Universitat Pompeu Fabra (UPF), 08003 Barcelona, Spain

Maintenance in restricted cold temperature conditions is a mandatory requirement to preserve the stability of mRNA vaccines. The latest update available for the COVID-19 vaccine manufactured by Moderna indicates that at a temperature of −20 °C is required for maintaining long-term stability for 6 months. In addition, Moderna has recently announced longer shelf life for its COVID-19 vaccine candidate at refrigerated temperatures [1], revealing that the stability could be maintained for 30 days between 2 °C and 8 °C. Any additional information that may be obtained about the maintenance conditions of these vaccines could be helpful for a better knowledge of these new products that are extremely useful to combat the COVID-19 pandemic.

On 22 April 2021, there was an accidental interruption in the cold chain storage of Moderna vaccine in the Pharmacy Department of Hospital del Mar when it was placed in a freezer programmed to maintain a temperature of −15 °C to −25 °C and controlled by the Sirius^®^ system. This system includes a software that controls the temperature of the refrigerator so that it is in the established range, as well as an alarm device when it detects that this variable is outside the established range. A temperature variation from −13.3 °C to −2.1 °C occurred in the freezer during a period of 5 h, reaching a maximum temperature of −2.1 °C for 20 min, but maintaining the temperature under −10 °C for a total period of 3 h. After this temperature disruption period, vaccines were maintained again at a temperature of −20 °C in the same freezer. This situation led to an immediate review of all control systems and an analysis of the stability of the available vaccines. Since there were seven boxes containing 10 vials each, it was decided to carry out the preparation of a sample of a syringe from one vial of each box, using at total of seven syringes as representative samples of the available boxes.

Moderna COVID-19 vaccines (0.5 mL) were prepared in a laminar flow chamber (Hospital del Mar Pharmacy Department) to be subjected to a stability analysis on mRNA integrity (Neuropharmacology-Neurophar and Genomics Core Facility). Microfluidic measurements to analyze mRNA integrity were performed using Agilent 2100 Bioanalyzer (Agilent technologies, Santa Clara, USA) with the RNA 6000 Nano LabChip kit and the assay Eukaryote total RNA Nano. Results were generated and analyzed with the Bioanalyzer 2100 Expert Software (Version B.0210.SI764). Smear analysis was used to define regions following the Bioanalyzer user guide. Our results reveal an absence of mRNA degradation in all the samples analyzed (Figure 1). Indeed, the RNA fractions area under the original mRNA peak was absent in all the cases. Any possible product of the degradation of the original mRNA should be identified in these fractions of lower molecular weight. We have previously obtained similar electropherograms with fresh samples of Moderna COVID-19 vaccines [2] to those shown in the present report, confirming the integrity of the currently analyzed vaccine samples.

Although mRNA COVID-19 Moderna unpunctured vials could be stable in standard refrigerators between 2° and 8 °C (36° to 46 °F) for 30 days [3] or 8° to 25 °C (46° to 77 °F) for a total of 24 h and between 2° to 25 °C (36° to 77 °F) after the first dose has been withdrawn for a total of 12 h [4], no information is available on the potential degree of affectation when exposed to unexpected changes in temperature, such as the one described here. The accidental interruption of the storage temperature conditions reported here had no consequences for the integrity of the mRNA contained in these Moderna COVID-19 vaccines. The accumulation of information on all aspects related to the packaging and handling of vaccines is of special importance, mainly in order to know the storage capacity of these vaccines in countries with limited resources that may be exposed to potential changes of storage temperature. Meanwhile, it is essential to establish the maximum control mechanisms recommended by the manufacturers of these vaccines in order to guarantee their stability and that their preventive activity is not altered by these factors.

## Figures and Tables

**Figure 1:**
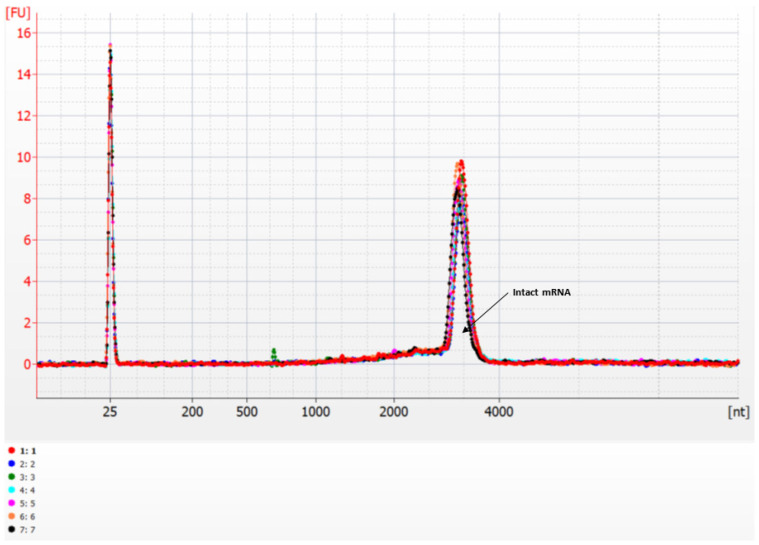
Analysis of mRNA integrity of reconstituted Moderna COVID-19 vaccines. Representative electropherograms expressed in fluorescence units (FU) of RNA integrity for 7 superposed different mRNA samples detailing the regions that indicate intact mRNA peaks in the analyzed samples.

## Data Availability

The data underlying this article will be shared on reasonable request to the corresponding author.
